# Transcriptional, post-transcriptional and chromatin-associated regulation of pri-miRNAs, pre-miRNAs and moRNAs

**DOI:** 10.1093/nar/gkv1354

**Published:** 2015-12-15

**Authors:** Chirag Nepal, Marion Coolen, Yavor Hadzhiev, Delphine Cussigh, Piotr Mydel, Vidar M. Steen, Piero Carninci, Jesper B. Andersen, Laure Bally-Cuif, Ferenc Müller, Boris Lenhard

**Affiliations:** 1 Biotech Research and Innovation Centre, University of Copenhagen, Ole Maaløes Vej 5, DK-2200 Copenhagen N, Denmark; 2 Zebrafish Neurogenetics Team, Paris-Saclay Institute of Neuroscience, CNRS UMR9197 – Université Paris Sud, 91198 Gif-sur-Yvette, France; 3 School of Clinical and Experimental Medicine, College of Medical and Dental Sciences, University of Birmingham, Edgbaston B15 2TT, UK; 4 Broegelmann Research Laboratory, Department of Clinical Science, University of Bergen, Bergen, Norway; 5 Malopolska Centre of Biotechnology, Jagiellonian University, Krakow, Poland; 6 Department of Clinical Medicine, University of Bergen, Norway; 7 Division of Genomic Technologies, RIKEN Center for Life Science Technologies, Yokohama, Kanagawa 230-0045, Japan; 8 Institute of Clinical Sciences MRC Clinical Sciences Centre, Faculty of Medicine, Imperial College London, Hammersmith Hospital Campus, Du Cane Road, London W12 0NN, UK; 9 Department of Informatics, University of Bergen, Thormøhlensgate 55, N-5008 Bergen, Norway

## Abstract

MicroRNAs (miRNAs) play a major role in the post-transcriptional regulation of target genes, especially in development and differentiation. Our understanding about the transcriptional regulation of miRNA genes is limited by inadequate annotation of primary miRNA (pri-miRNA) transcripts. Here, we used CAGE-seq and RNA-seq to provide genome-wide identification of the pri-miRNA core promoter repertoire and its dynamic usage during zebrafish embryogenesis. We assigned pri-miRNA promoters to 152 precursor-miRNAs (pre-miRNAs), the majority of which were supported by promoter associated post-translational histone modifications (H3K4me3, H2A.Z) and RNA polymerase II (RNAPII) occupancy. We validated seven miR-9 pri-miRNAs by *in situ* hybridization and showed similar expression patterns as mature miR-9. In addition, processing of an alternative intronic promoter of *miR-9–5* was validated by 5′ RACE PCR. Developmental profiling revealed a subset of pri-miRNAs that are maternally inherited. Moreover, we show that promoter-associated H3K4me3, H2A.Z and RNAPII marks are not only present at pri-miRNA promoters but are also specifically enriched at pre-miRNAs, suggesting chromatin level regulation of pre-miRNAs. Furthermore, we demonstrated that CAGE-seq also detects 3′-end processing of pre-miRNAs on Drosha cleavage site that correlates with miRNA-offset RNAs (moRNAs) production and provides a new tool for detecting Drosha processing events and predicting pre-miRNA processing by a genome-wide assay.

## INTRODUCTION

MicroRNAs are a class of small (∼22 nucleotide (nt)) regulatory non-coding RNAs found in plants and animals. These small RNAs are processed from large miRNA primary transcripts (pri-miRNAs) into 70∼90 nt precursors (pre-miRNAs) and further into mature miRNA (1,2). Pri-miRNAs are generally transcribed by RNA polymerase II (RNAPII) (3), although a few miRNAs are transcribed by RNAPIII (4). Rapid processing and relatively low copy number have hindered identification and characterization of pri-miRNA, limiting the studies to only a handful of examples (3,5,6). However, high throughput sequencing technologies have facilitated annotation of pri-miRNAs on a genome-wide scale in human and mouse (7–12). Pri-miRNA promoters are located anywhere from about a hundred of bases to several kilobases (KB) upstream of the pre-miRNA. Initial studies detected independent promoters only for a few intronic pre-miRNAs (i.e. miRNAs whose pre-miRNA is found in the intron of known protein-coding genes) (7–11). However, a subsequent study showed many intronic pre-miRNAs have independent promoters that exhibit tissue-specific regulation (13). Interestingly, some pre-miRNA can also be transcribed locally by transcription initiation at their 5′-ends (∼50 bases from mature miRNA) (14).

Transcription of pri-miRNA transcripts and pre-miRNA cleavage are nuclear events that are facilitated by microprocessor components Drosha/DGCR8 (15). It has been proposed that pre-miRNAs are processed co-transcriptionally (16,17). The details of targeting and regulation of this cleavage are still unclear, although it is believed to involve regulatory information embedded in the primary transcripts (16). Flanking single-stranded RNA segments located outside the pre-miRNA hairpin are crucial for precise cleavage of the stem loop and maturation of miRNA (18,19). Coupling between pri-miRNA transcription and Drosha processing promotes efficient expression of transcribed miRNAs (20). Sequence motifs associated with co-transcriptional cleavage remain elusive, thus it may be mediated via interplay between transcription and chromatin regulation. Genome-wide analysis of nucleosome positioning has revealed nucleosomes are positioned along pre-miRNAs (8). Analyses of pre-miRNA processing have revealed a distinct class of small RNAs (miRNA-offset RNAs (moRNAs)) originated from the sequence flanking 5′- or 3′- arms of pre-miRNAs (21,22). Although the exact biogenesis of moRNAs remains unclear, they might represent RNA products generated by Drosha during pre-miRNA processing (21).

The prediction of pri-miRNA promoters remains notoriously difficult due to the rapid and efficient nature of Drosha/DGCR8 processing. Though pri-miRNA promoters have been mapped in human and mouse to various extents (7–13), in zebrafish—an important vertebrate model organism—pri-miRNA promoters remain largely unannotated. The availability of comprehensive annotation of zebrafish pri-miRNAs will provide a valuable resource to study mechanistic insights on pri-miRNA regulation during embryonic development. Recent findings report the existence of both distal promoters on host genes and local promoters at pre-miRNAs (14), suggesting alternative mechanisms of how miRNAs may be produced. CAGE-seq technology can detect primary and alternative promoters (23,24) and also detect a variety of processed RNAs (24,25). In this paper, we explored the utility of this technology to study transcriptional and post-transcriptional processing of pri-miRNAs during development. We have comprehensively annotated zebrafish pri-miRNAs transcripts and unraveled processing events and their utilization during early embryogenesis. We used CAGE-seq defined transcription start site (TSS) maps at single nucleotide resolution to identify pri-miRNA promoters. Furthermore, we demonstrated that our predicted pri-miRNAs are *bona fide* transcripts by validating seven paralogous copies of miR-9, by describing their processing and by identifying their expression patterns. Moreover, we show that promoter-associated H3K4me3, H2A.Z and RNAPII marks are not only present at pri-miRNA promoters but are also specifically enriched at pre-miRNAs, suggesting chromatin level regulation of pre-miRNAs. We report novel insight about post-transcriptional processing of pre-miRNA hairpins that correlate with the presence of miRNA-offset RNA production.

## MATERIALS AND METHODS

### Data sets

Processed CAGE tags from 12 developmental stages, RNA-seq data from four developmental stages and post-translation histone modification data (H3K4me3 (four developmental stages) and H2A.Z) were produced in our recent study (24,26). Raw sequence reads of RNAPII (27) and small RNA-seq data (28) were downloaded from SRA. Detailed information regarding mapping and statistics of analyzed data is described in Supplementary Table S1. Sequence reads were mapped to the zebrafish genome assembly (Zv9). The genomic coordinates of 346 pre-miRNA hairpins were downloaded from miRBase-V20 (29). We excluded 25 unexpressed pre-miRNAs and retained 321 pre-miRNAs that are expressed above 1 tag per million (tpm). Based on Ensembl annotation (coding and non-coding host genes), overlapping pre-miRNAs were classified as intragenic, and remaining 217 (including 59 copies of miR-430 in cluster) pre-miRNAs as intergenic. Ensembl annotated pre-miRNAs (30) were downloaded, and we excluded pre-miRNAs residing on coding exons and those annotated in miRBase-V20, leaving 227 pre-miRNAs. CpG islands (CGIs) were downloaded from UCSC genome browser database (31) and in-house custom annotated CGIs. Mapped small RNA and histone modification (aligned BAM files and peak calling) data from h1-ESC cell lines were downloaded from ENCODE (32).

### CAGE transcription start sites

A CAGE tag mapped to the genome is called CAGE transcription start site (CTSS). The first base of CAGE tag defines the TSS and the number of CAGE tags mapped to that nucleotide gives quantitative measure of CTSS, which is measured as tpm. Proximal CTSSs within 20nt apart, on the same strand, are clustered as transcript clusters (TCs) (Supplementary Figure S1A), where TSS with highest expression values defines the representative CTSSs. The width of TC and distribution of CAGE tags is used to classify sharp and broad promoters. Initiator sequence is defined by the first base (+1) of CAGE tag and the immediate upstream base (−1). Canonical initiators are denoted by pyrimidine and purine at −1, +1 position. A class of noncanonical initiators associated with post-transcriptional processing are denoted by ‘Guanine’ at −1, +1 position (24). CAGE TCs were further classified as transcriptional-associated and post-transcriptional associated based on initiator sequence. We measured the expression level of pri-miRNAs by summing the expression of all CAGE TCs in the defined promoter region (±500 bases).

### Identification of pri-miRNA promoters

We systematically analyzed all CAGE TCs (≥0.5 tpm) in the upstream regions (up to 40 kB) of intergenic pre-miRNAs, iteratively by increasing upstream distance with step size of 5 kB. However, if annotated coding genes are encountered within 40 kB, the upstream region was extended up to 3′-ends of genes. We first compiled all CAGE TCs in the assigned upstream region and excluded post-transcriptionally associated CAGE TCs. On remaining CAGE TCs, we ensured the immediate upstream CAGE TCs were not annotated (by default) as pri-miRNA promoters. We analyzed all CAGE TCs in the next upstream regions and prioritized their association with pre-miRNAs based on RNA-seq/EST and H3K4me3 evidence. If only a single CAGE TC was detected, it was assigned as pri-miRNA promoter. If multiple CAGE TCs were detected, we used RNA-seq/EST transcripts to determine the CAGE TCs that are associated with full-length transcripts or have concordant 5′-ends. CAGE TCs without evidence of RNA-seq/EST transcripts were searched for the presence of enriched H3K4me3 signals. CAGE TCs without RNA-seq/EST and H3K4me3 evidence were prioritized based on high expression level of CAGE TCs. Pre-miRNAs without any assigned promoter within 40 kb upstream were excluded as undetectable annotated pri-miRNAs. After annotation of pri-miRNAs, we analyzed intronic CAGE TCs to identify alternative promoters, where such CAGE TCs had to be at least ±500 bases away from assigned pri-miRNAs. For intragenic pre-miRNAs residing on a non-coding host, the annotated transcripts were assigned as pri-miRNA transcripts. For pre-miRNAs residing on a coding host, upstream regions were extended up to the 5′-ends of host genes, and examined for evidence of independent transcription that are distinct from host promoters. For pre-miRNAs with undetected alternative promoters, it remains unclear whether they are co-transcribed by host genes or have independent promoters that remain undetected in our datasets. However, if pre-miRNAs are located close (<5 kB) to the 5′-ends of host genes, and had no evidence of independent transcription, we considered them to be transcribed by host gene, resulting in 13 such pre-miRNAs.

### Micro-RNA offset RNAs

Micro-RNA offset RNAs (moRNAs) are generated adjacent to 5′ arm and 3′ arm of mature miRNAs. We intersected CAGE tags with respect to annotated pre-miRNAs and identified a subset of pre-miRNAs that have CAGE tags. To define a CAGE-based moRNA, we ensured that CAGE tags originated adjacent (1 base offset) to the mature miRNAs, were detected in at least two developmental stages and transcribed from the same nucleotide.

### Bioinformatics tools and analyses

All bioinformatics analyses were performed using Perl, R/Bioconductor, bash scripts and Bedtools suite (33). Sequence logos were drawn using WebLogo (34). Small RNA-seq reads were mapped using bowtie (35) with default parameters.

### RNA extraction, *in situ* hybridization and RACE PCR

Total RNA from 24 h post-fertilization (hpf) embryos was extracted using Trizol (Ambion), treated with DNase I (Promega) and reverse transcribed with Superscript II reverse transcriptase (Invitrogen) using random hexamer primers. Seven paralogous copies of assembled primary transcripts (described below) of miR-9 were amplified by PCR using the following primers (Supplementary Table S2), and cloned using Strataclone PCR cloning kit (Agilent technologies). 5′ RACE PCR experiments were performed using the 5′ RACE system for rapid amplification of cDNA ends (Invitrogen), using the listed primers (Supplementary Table S2). Antisense DIG labeled RNA probes for each miR-9 transcripts were synthesized and *in situ* hybridization performed using standard protocols (36).

## RESULTS

### High throughput identification of pri-miRNA promoters

**Figure 1.**
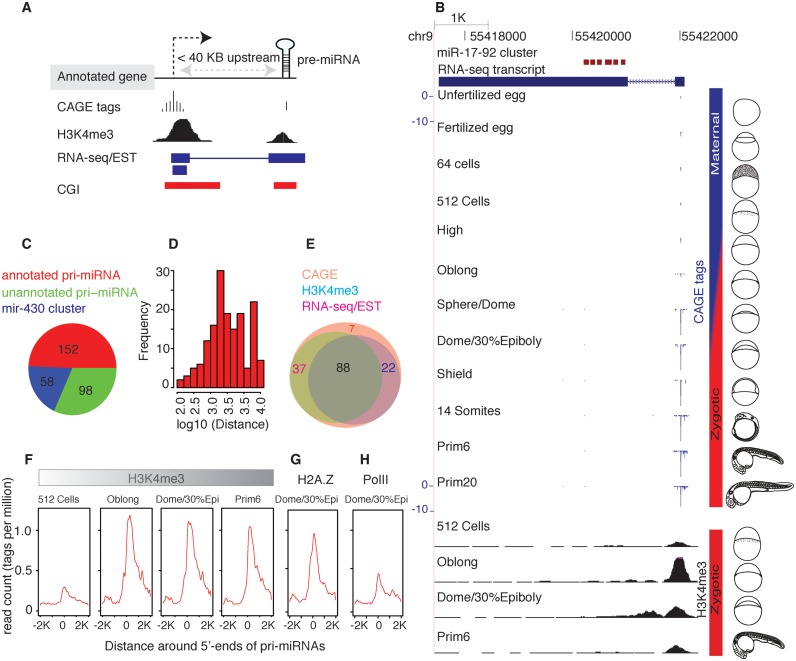
Mapping of zebrafish miRNA primary transcripts during embryonic development. (**A**) Schematic representation of the approach used to predict pri-miRNA transcripts (see Materials and Methods). (**B**) A genome browser view of pri-miRNA transcript of intergenic miR-17–92 cluster. Full-length transcript is reconstructed from RNA-seq and the promoter transcription start site is defined by CAGE-seq. H3K4me3-modified nucleosomes at 5′-ends provides additional evidence for being a true promoter. Maternal and zygotic transcriptome stages are represented by vertical bars in blue and red, respectively. (**C**) Distribution of pre-miRNAs with annotated pri-miRNAs (red), other pre-miRNAs without annotated pri-miRNAs (green) and miR-430 pre-miRNAs (blue). (**D**) Distance of pri-miRNA TSSs relative to pre-miRNA. (**E**) Number of pri-miRNAs TSSs defined by CAGE-seq that are supported by H3K4me3 peaks and RNA-seq/EST transcripts. (**F–H**) Alignment of promoter-associated histone modifications (H3K4me3, H2A.Z) and RNAPII supports the annotated pri-miRNAs 5′-ends. Y-axis shows the normalized read count in tags per million (tpm).

To annotate TSSs of zebrafish pri-miRNA promoters, we systematically analyzed CAGE tags in the upstream region (see Materials and Methods) of 321 pre-miRNAs that are expressed during early embryogenesis. Adopting the criteria from previous studies (7,8,13), we analyzed up to 40 kB upstream of pre-miRNAs as the likely region to encode pri-miRNAs (Figure 1A). We identified pri-miRNA promoters for 152 (92 intergenic and 60 intragenic) pre-miRNAs in the assigned upstream region (Supplementary Table S3). Out of 60 intragenic pre-miRNAs, 31 pre-miRNAs reside on annotated non-coding host transcripts and the remaining 29 pre-miRNAs are inside protein coding genes. Out of these 29 pre-miRNAs, we identified independent promoters for 16 pre-miRNAs, while the remaining 13 pre-miRNAs lie proximal to 5′-ends of protein coding genes; thus, host gene promoters were annotated as the pri-miRNA promoter. As an illustrative example, primary transcript of the intergenic miR-17–92 cluster is shown with full-length transcript, CAGE tags and H3K4me3 peaks (Figure 1B). CAGE-seq defined TSS is located 1.1 KB upstream from the first pre-miRNA, where the representative TSS is determined by the position of dominant CAGE tags within the tag cluster (Supplementary Figure S1A). Six pre-miRNAs of the miR-17–92 cluster are encoded by a single primary transcript, as reported in human (6). Counting the 59 copies of miR-430 in a cluster as a single gene, we annotated pri-miRNA TSS for ∼58% of total pre-miRNAs (Figure 1C). We were unable to detect pri-miRNA promoters for 55 intergenic pre-miRNAs (excluding the miR-430 cluster) and 42 intronic pre-miRNAs residing in protein coding host genes. Lack of evidence for independent transcription of intragenic pre-miRNAs suggests that they are either co-transcribed with their host genes or, alternatively that intragenic pre-miRNAs might have tissue specific pri-miRNAs (13) that remain undetected in the analyzed datasets. However, this cannot be confirmed without additional data. The lack of pri-miRNA TSSs for 56 intergenic pre-miRNAs might be due to one of several reasons: (i) tissue-specific or low expression that remains undetectable in our dataset or (ii) transcribed by PolIII (4) that is undetected by CAGE-seq. Even so, the depth of the annotated pri-miRNAs is comparable to previous studies in human and mouse (7,8). We also analyzed the region between the annotated pri-miRNA promoters and pre-miRNAs for potential alternative promoters, and identified additional CAGE TCs associated with 47 pre-miRNAs (Supplementary Table S3), but those TCs had low expression levels compared with the main identified promoter. The TSSs of predicted pri-miRNAs are located from a few hundred bases up to tens of kB upstream of pre-miRNAs (Figure 1D), similar to previous observations in human and mouse (7,8). The majority of annotated pri-miRNAs promoters have additional evidence of promoter activity from complementary genome-wide data (RNA-seq/EST and H3K4me3) (Figure 1E). Pri-miRNA promoter regions of 123 pre-miRNAs had concordant 5′-ends with RNA-seq/EST (87 and 118 respectively) transcripts and 109 pre-miRNAs had robust H3K4me3 peaks (Figure 1E). Promoter-associated signals such as H3K4me3-modified nucleosomes, histone variant H2A.Z and RNAPII occupancy are all enriched directly downstream of aggregated predicted pri-miRNA 5′-ends (Figure 1F–H), further supporting prediction of genuine promoters by CAGE-seq.

### Validation of pri-miRNAs transcription start site regions and pre-miRNA processing sites of the miR-9 family

To validate the annotated pri-miRNA promoters, we selected the miR-9 family with seven paralogous copies. The miR-9 family is highly conserved and a crucial regulator of embryonic neurogenesis in vertebrates (37). In zebrafish embryos, miR-9 expression is detected around 20 hpf (30 somites stage) in the telencephalon, and later spreads to a more posterior region of the brain (38). Distinct CAGE tags mapped to the promoter of annotated pri-miRNA transcripts of miR-9 (Supplementary Figure S2). Except for *miR-9-1*, CAGE-seq detected miR-9 pri-miRNA TSSs at 16 hpf (14 somites stage) (Supplementary Figure S3A) before mature miR-9 becomes detectable by *in situ* hybridization. In addition to the host promoter, we detected an alternative intronic promoter by CAGE in the upstream (∼1.1 kB) region of *miR-9-5* (Supplementary Figure S2). Notably, the alternative intronic TSS and host transcript TSS of *miR-9-5* are simultaneously initiated at the 14 somites stage as detected by CAGE-seq and validated by 5′ RACE PCR.

**Figure 2.**
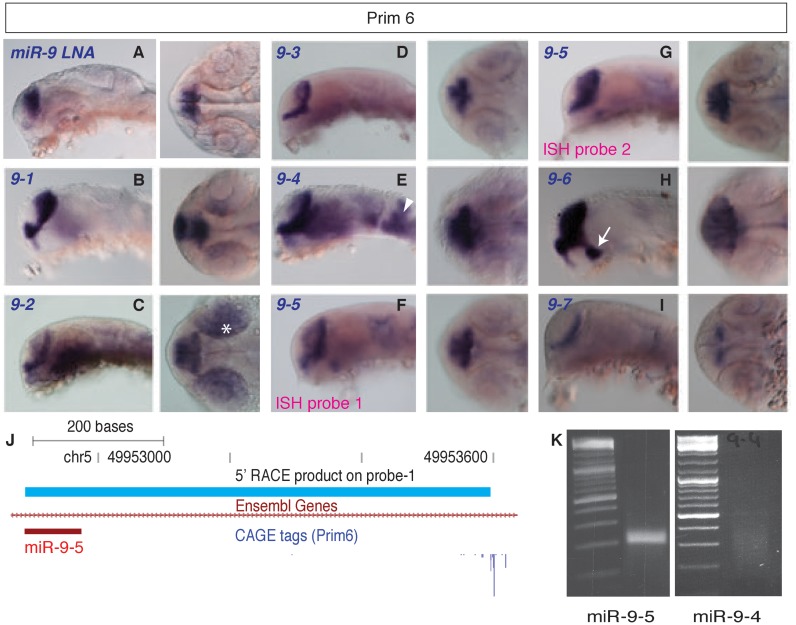
Expression pattern revealed by *in situ* hybridization of mature miR-9 and pri-miRNA transcripts during prim6 stage. (**A**) Expression pattern of mature miR-9 is mostly restricted to telencephalon. Left panels represent lateral views and right panels represent dorsal views. (**B–I**) Expression pattern of miR-9 pri-miRNA transcripts is also restricted to telencephalon with differences in the spatiotemporal details among different pri-miRNAs, such as: *miR-9-2* (developing retina, white asterisk; Figure 2C), *miR-9-4* (posterior brain regions, white arrowhead; Figure 2E) and *miR-9-6* (hypothalamus, white arrow; Figure 2H). (**J**) Amplified region of 5′ RACE PCR product (corresponding to probe-1) of *miR-9-5* maps exactly to the alternative intronic CAGE tags. (**K**) Unique discrete amplification band obtained from 5′ RACE PCR products obtained for *miR-9-5* (with intronic CAGE tags). Multiple non-specific bands and/or a smear were obtained for *miR-9-4* that did not have alternative intronic CAGE tags.

The expression pattern of mature miR-9, determined by miR-9 antisense LNA probe, is restricted to telencephalon during prim6 stage (Figure 2A). Spatiotemporal distribution of each miR-9 pri-miRNA was determined by ISH probes (Supplementary Figure S3B), and was highly reminiscent of the mature miR-9 LNA probe, mostly restricted to the telencephalon at prim6 (Figure 2B-I) and at later stages including the posterior brain (Supplementary Figure S3C), thus validating these transcripts as miR-9 precursors. However, differences in fine patterns of expression were detected between different miR-9 family members most notably at prim6. For instance, *miR-9-2* is highly expressed in the developing retina (Figure 2C, white asterisk), *miR-9–4* showed early expression in the posterior brain regions (Figure 2E, white arrowhead) and *miR-9-6* showed a distinctive strong expression in the hypothalamus (Figure 2H, white arrow). Expression of these pri-miRNA precedes the appearance of detectable levels of mature miR-9 in these brain regions, as seen at 48hpf (Supplementary Figure S3C). The differential distribution of miR-9 pri-miRNAs might generate different level of mature miR-9 in different brain areas relative to its mRNA targets, and might thus impact its functional output. In contrast, the alternative intronic TSS (probe-1) and host transcript TSS (probe-2) of *miR-9-5* pri-miRNA showed an identical spatial distribution (Figure 2F and G).

To address if the observed intronic CAGE tags of *miR-9-5* acts as an alternative promoter, we performed 5′ RACE PCR (primers located upstream of pre-miRNA (see Materials and Methods; Supplementary Table S2)) on *miR-9-5* and compared it to *miR-9-4* in which no alternative intronic CAGE tags were detected. 5′ RACE product of *miR-9-5* extended exactly to the intronic CAGE tags (Figure 2J and K), thus confirming an alternative usage of intronic promoter that is independent of its host transcript. In contrast, 5′ RACE PCR of *miR-9-4* did not reveal any discrete TSS in the upstream region (Figure 2K) consistent with the lack of alternative intronic CAGE tags, further suggesting that *miR-9-4* is processed from its host gene as predicted by CAGE analysis.

### Characteristic features of pri-miRNA promoters

**Figure 3.**
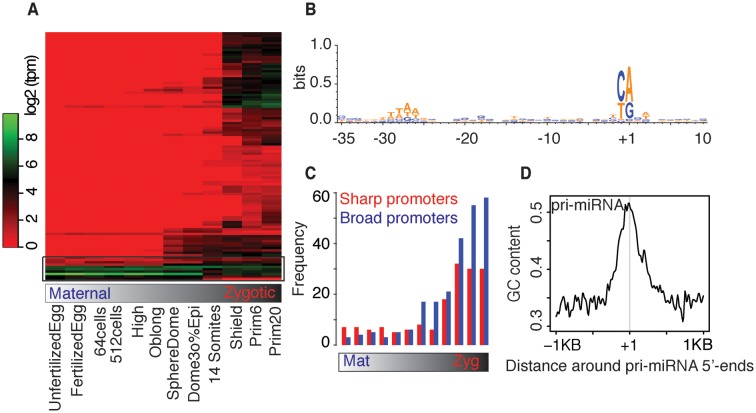
Developmental dynamics of pri-miRNAs and their characteristics features. (**A**) Clustering of pri-miRNAs based on the activity of annotated promoters across 12 developmental stages. The heat map represents the expression level (log_2_(tpm)) at indicated stages. Black rectangle indicates maternally inherited pri-miRNAs. (**B**) Alignment of sequences based on representative transcription start sites of pri-miRNAs reveal an enrichment of canonical initiators and associated TATA-like motifs. (**C**) Distribution of sharp and broad promoters and their dynamic usage across developmental stages. X-axis indicates 12 developmental stages, same as in (**A**). (**D**) Average G+C content of pri-miRNAs promoters. Y-axis indicates the average GC content.

Next we computed the expression level of pri-miRNA promoters (see Materials and Methods) and investigated their dynamics during early embryogenesis by hierarchical clustering (Figure 3A). Only 12 pri-miRNAs (encoding 19 pre-miRNA; Supplementary Table S3) are maternally inherited and remain active (except for miR-30a) during the critical phase of vertebrate ontogeny. Pri-miRNAs of miR-30a is active only during maternal stages and no zygotic transcription was detected within the stages examined. The majority of remaining pri-miRNAs are zygotically initiated. Compared to coding genes, pri-miRNAs are expressed at low levels (Supplementary Figure S4A), which in addition to their rapid processing may be due to the cell/tissue-specific activity of miRNAs (39,40), which is diluted in whole-embryo RNA libraries.

Alignment of representative TSSs of pri-miRNAs revealed an enrichment of canonical initiators (Figure 3B), similar to genome-wide enrichment of promoters (23,24). TATA-like motifs enriched in upstream regions were mostly non-canonical TATA-like elements (W-box motifs as described in (26)) as only 9 pri-miRNAs had a canonical TATA box (characterized by TATAWA). We further classified the core promoters into ‘sharp’ and ‘broad’ promoters (Supplementary Figure S4B) (23) and observed a preference for broad promoters (Figure 3C), although sharp promoters are preferred during early (maternal transcriptome) stages, similar to the genome-wide patterns (24,26). Sharp promoters are enriched for W-box when compared to broad promoters (Supplementary Figure S4C) as expected (26). Pri-miRNAs promoters have high GC content centered around their 5′-ends (Figure 3D) where 54 (51.4%) pri-miRNA promoter regions overlap with CGIs.

### Promoter-associated histone modifications and PolII are enriched on pre-miRNAs

**Figure 4.**
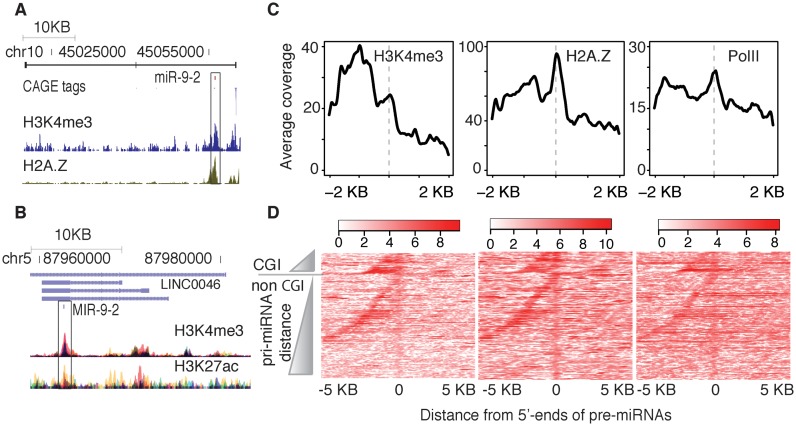
Promoter-associated histone modifications at pre-miRNAs differ from those at pri-miRNA promoters. (**A**) A genome browser view of zebrafish *mir-9–2* with CAGE tags, H3K4me3 and H2A.Z tracks. H3K4me3 and H2A.Z peaks at pre-miRNA are distinct from pri-miRNA. (**B**) A genome browser view of human orthologous *MIR-9-2* with H3K4me3 and H3K27ac tracks. (**C**) Alignment of average H3K4me3 (prim6 stage), H2A.Z and RNAPII (both Dome/30%Epiboly stage) marks along the 5′-ends of pre-miRNAs reveal an enriched peak associated with pre-miRNAs. (**D**) Heat maps showing H3K4me3, H2A.Z and RNAPII signals of each pre-miRNAs. Pre-miRNAs overlapping CGIs are stacked on top and pre-miRNAs not overlapping CGIs are stacked below in the increasing order of pri-miRNA distance.

It was recently demonstrated that miR-320a is directly transcribed at the 5′-end of the pre-miRNA, suggesting an alternative transcriptional mechanism for the generation of miRNAs (14). We have noticed H3K4me3-modified histones are present on pre-miRNA sites of individual miR-9 in zebrafish and human (Figure 4A,B; Supplementary Figure S2). Motivated by these observations we analyzed chromatin features and CAGE signals on pre-miRNAs on a genome-wide scale. We aligned promoter associated H3K4me3, H2A.Z and RNAPII marks along 5′-ends of pre-miRNAs and observed an overall enrichment (Figure 4C). Closer inspection at the individual pre-miRNAs revealed around 47% (71 out of 152) of pre-miRNAs show enriched H3K4me3 peaks (Supplementary Table S3). Similarly, alignment of H3K4me3, H2A.Z, H3K27ac and H3K9ac modified histones along 5′-ends of pre-miRNAs in human embryonic stem cells (hESC) revealed an enriched peak (Supplementary Figure S5A-D). Moreover, pre-miRNA sites overlapping with CGIs carry higher level of histone modifications (Supplementary Figure S5E-H). We identified 77 (out of 346 expressed in hESC) pre-miRNAs had enriched H3K4me3 peaks, of which 19 had enriched H3K4me3 peaks in both zebrafish and human (Supplementary Table S4), indicating that it is an evolutionarily conserved feature.

These promoter-associated features detected at pre-miRNAs may be due to a pre-miRNA specific chromatin modification, but could also be due to overlap with the extended region originating from the host gene promoter and running into the body of the gene for several kilobases, as observed in developmental regulator genes (41,42). To test if the enrichment of promoter-associated chromatin features is pre-miRNA specific, we aligned individual pre-miRNAs according to increasing distance from their pri-miRNA start sites. This analysis revealed that modified histones can be aligned separately on pre-miRNAs from their pri-miRNA promoter regions (Figure 4D), suggesting that both pre-miRNA and pri-miRNA start regions can be independently marked by promoter-associated modified histones and RNAPII. Out of 71 pre-miRNAs, 28 pre-miRNAs had independent H3K4me3 peaks that were disjointed from H3K4me3 peaks associated with pri-miRNA. The remaining 43 pre-miRNAs had H3K4me3 peaks that spanned from pri-miRNA to pre-miRNA and had different enrichment patterns (Supplementary Figure S5I). Since pre-miRNAs are spliced RNAs, specific enrichment of promoter-associated modified histones on a subset of pre-miRNAs was rather unexpected and could represent either (i) a local promoter undetectable by CAGE-seq (similar to the only known miRNA with proximal TSSs (14)) or (ii) the involvement of chromatin regulation in co-transcriptional processing of pre-miRNAs (16).

### Detection of Drosha processing sites for miRNA-offset RNA (moRNA) by CAGE

**Figure 5.**
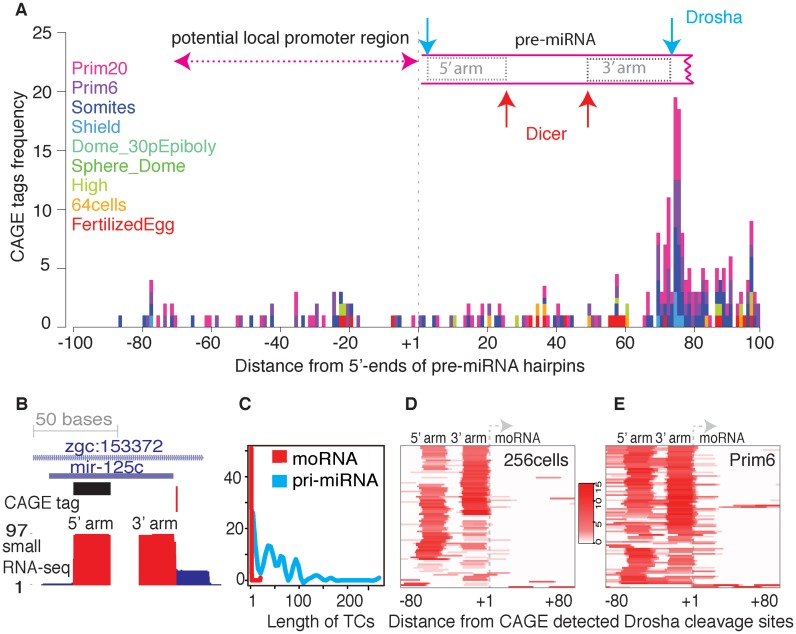
CAGE-seq detects post-transcriptional processing of pre-miRNAs on Drosha cleavage sites. (**A**) Alignment of CAGE tags around pre-miRNAs reveal post-transcriptionally generated CAGE tags are enriched at Drosha cleavage site during zygotic stages. Pre-miRNAs are schematically represented by rectangular boxes. Broken edges towards 3′-end represents variable length. Red and blue arrows indicate the Dicer and Drosha cleavage sites. (**B**) A genome browser view of *miR-125c* with CAGE tags and small RNA tracks from prim6 stage. Black horizontal bar represent annotated mature miRNA. Small RNA reads map to mature miRNA arms and flanking regions (blue blocks). (**C**) Comparison of length of transcription initiation clusters (TCs) of moRNAs and pri-miRNAs. (**D** and **E**) Small RNA reads aligned to 5′-ends of Drosha cleavage sites detected by CAGE-seq from (**D**) maternal (256 cells) and (**E**) zygotic (Prim6) transcriptome revealed that moRNA production occurs predominantly in zygotic transcription.

To further analyze the transcriptional and post-transcriptional features of pre-miRNAs, we aligned CAGE tags around 5′-ends of pre-miRNAs. We detected distinct enrichment of CAGE tags at the 3′-ends of pre-miRNAs (Figure 5A). Upon inspection of CAGE tags mapped to the loci of pre-miRNA hairpins genome wide, we observed that they are often present adjacent to the 3′-end of these hairpins (Figure 5B; Supplementary Figure S6A–C), thus confirming that CAGE tags map preferentially to the nucleotide immediate downstream of mature miRNA. The CAGE tags were detected at 3′-end of pre-miRNAs in 61 known pre-miRNA genes (Supplementary Table S5) and detected only during zygotic stages. The underlying sequences do not correspond to TSSs (Supplementary Figure S6D) and the signal is very ‘sharp’ (no spreading, Figure 5C). These observations suggest that the detected RNA represents Drosha cleavage product detected by CAGE-seq, similarly to other post-transcriptionally processed RNA products (24,25), and corresponds to 5′-end of the so-called miRNA-offset RNAs, detected previously in in *Ciona intestinalis* (21) and mammals (22).

To validate that the presence of CAGE tags at moRNA precursor sites is due to the presence of moRNAs themselves, we analyzed small RNA libraries generated from maternal (256 cells) and zygotic (prim6) transcriptomes (see Materials and Methods). We identified 69 pre-miRNAs with moRNAs (50 on the 5′ arm and 35 on the 3′ arm; 16 pre-miRNAs have moRNAs on both arms) by using at least 1 read per site as the threshold. By increasing the minimum threshold to 2, 46 pre-miRNAs with moRNAs are detected (33 on 5′ arm and 21 on 3′ arm; 8 pre-miRNAs have moRNAs on both arms) (Supplementary Figure S6E). The low expression of moRNAs is consistent with previous reports (21,22), and partly explains why it is difficult to uncover such events unless sequenced at unprecedented depth. Comparison of moRNAs detected by CAGE-seq and by small RNA-seq revealed only 29 moRNAs detected by both techniques. Small RNAs are produced in either of the arms (Figure 5D and E; Supplementary Figure S6A–C) with slight preference for the 5′ arm. However, during 256-cells (maternal) stage only four pre-miRNAs had moRNAs (≥2 reads), and no moRNAs were detected by CAGE-seq. This suggests that moRNAs are primarily zygotically initiated and are *de novo* processed transcripts rather than inherited from the oocyte.

### moRNA CAGE as predictive tool for the validation of miRNA discovery

The results presented above indicate that moRNA-associated CAGE signal is a predictor of actively processed pre-miRNA hairpins. To test if CAGE-seq could be used as a tool for biochemical validation of computationally predicted pre-miRNA hairpin processing, we analyzed 227 predicted pre-miRNAs (see Materials and Methods). We identified CAGE tags only on four pre-miRNAs where only a single pre-miRNA had evidence with high confidence of active Drosha processing (Supplementary Figure S6F). Furthermore, we also annotated its pri-miRNA promoter and observed enriched H3K4me3 modified nucleosomes along pre-miRNAs. The drastically lower rate of active processing of pre-miRNAs observed on computationally predicted pre-miRNAs suggests that the large majority of computationally predicted miRNAs may not be biologically functional, and that moRNA detection provides strong independent evidence for their function.

### Validation of CAGE detected moRNAs processing sites by RACE PCR

**Figure 6.**
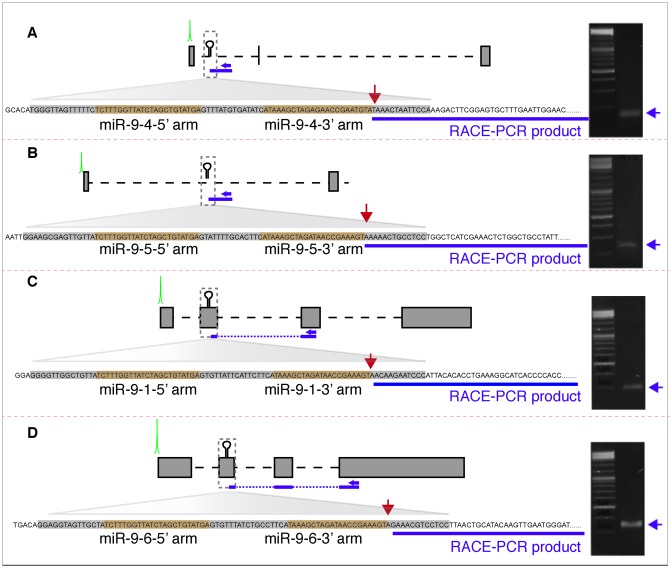
3′ RACE PCR amplification of miR-9-(1/4/5/6). (**A–D**) Schematic representations of miR-9-(1/4/5/6) loci and designed primers based on downstream sequences (see Supplementary Table S3). The resulting 3′ RACE PCR products are shown in dark blue. Mature miRNAs sequences are highlighted in orange and the remaining sequences of pre-miRNA hairpins in grey. 5′-end of RACE PCR products (blue bars) precisely mapped Drosha cleavage site (red arrow). The agarose gel images on the right show that unique RACE PCR products were obtained.

We next sought to ask if CAGE tags detected on pre-miRNAs can be verified by an alternative 5′ cap detection method. We performed 3′ RACE PCR experiments using primers located downstream of pre-miRNA (see Materials and Methods) on the predicted primary transcripts of *miR-9-4/5* (located on intron of host gene) and *miR-9-1/6* (both of which harbor an exonic hairpin) (Figure 6). Analysis of *miR-9–4/5* products indicated that the 5′-ends mapped exactly to the annotated Drosha cleavage site (red arrow; Figure 6A and B), further suggesting that it is first processed from the host intron and subsequently capped. Similarly, analysis of *miR-9–1/6* products indicated that the 5′-ends mapped in close vicinity (1 base offset) to the predicted Drosha cleavage site (red arrow; Figure 6C and D). Interestingly, *miR-9-1*/*6* products correspond to the spliced form of their primary transcript, suggesting that processing by Drosha occurred downstream of primary transcript splicing. Consistently, unprocessed spliced pri-miRNAs of *miR-9-1/6* can be amplified by RT-PCR, as was demonstrated when we cloned *miR-9-1* and *miR-9-6* ISH probes (Supplementary Figure S3B).

## DISCUSSION

We have presented a comprehensive annotation of zebrafish pri-miRNA promoter repertoires that can serve as a valuable resource for the study of miRNA-mediated regulatory networks. Overall, the annotation shows that pri-miRNA promoters show both the sequence properties and functional diversity comparable to those of protein-coding genes. Different pri-miRNA promoters have enriched GC content (often overlapping CGIs) and many also have TATA-like motifs, as well as combinations of features characteristic for the promoters of genes under long-range developmental regulation (41). This suggests that there is no mode of transcriptional regulation specific for pri-miRNA genes.

One notable observation from the analysis is the presence of potential maternally inherited pri-miRNAs during development of the zebrafish embryo. While we detected 12 pri-miRNAs as maternally inherited, however, post-transcriptionally associated CAGE tags on Drosha processing sites were not present on them during maternal stages, but were detected only after mid-blastula transition. This suggests that maternally deposited pri-miRNAs could require a zygotically activated regulator for processing pre-miRNAs from them. To characterize potential functions of maternally inherited pri-miRNAs further experiments are required, such as the recent study reporting unique mechanism of adenylation of maternally inherited miRNAs (43). However, the maternally inherited miR-17–92 cluster, which has similar seed sites as miR-430 and human embryonic regulator miRNA (miR-302/miR-467) (44), may be of great importance to developmental biologist in studying their potential roles in degradation of maternal transcripts during early embryogenesis.

Enrichment of promoter-associated modified histones and RNAPII signals on pre-miRNAs raises an interesting question on whether they reflect an overlap with a known chromatin feature of genes under long-range developmental regulation, or represent an additional layer of chromatin regulation involved specifically in co-transcriptional processing of pre-miRNAs. Based on the results from our own analysis and previous studies, we have evidence supporting both scenarios on various pre-miRNAs. We detected specific enrichment of H3K4me3, H2A.Z and RNAPII peaks at pre-miRNAs suggesting local promoter activity, however CAGE-seq and 5′ RACE PCR of two pre-miR-9 candidates did not support production of capped RNA at these predicted local promoter regions (Figure 2J and K). Similarly, 3′ RACE PCR on four pre-miR-9 candidates revealed no local promoter activity, but indication of Drosha cleavage. These evidence collectively argues against activity of local promoters at pre-miRNAs. Alternatively, the chromatin features raise the possibility of a yet unexplained chromatin mediated mechanism for a co-transcriptional processing of pre-miRNAs produced from a distal pri-miRNA promoter, similar to chromatin association of transcription and mRNA splicing (45,46). A new study using a different sequencing technique (native elongation transcript; NET-seq) in human cell lines showed nascent transcripts mapped at the expected Drosha cleavage sites that are associated with co-transcriptional processing of pre-miRNAs (47). Furthermore, DNA sequences encoding mature miRNAs were preferentially occupied by positioned nucleosomes (8), which may be associated with co-transcriptional pre-miRNA cleavage. Such a scenario may partly explain why miRNA-encoding introns are spliced at a slower rate than adjacent introns (48).

Although our understanding of moRNA biogenesis and function remains lacking, it has been speculated that moRNA biogenesis might be associated with pri-miRNA processing. If so, it is unclear why moRNAs are detected only on subset of pre-miRNAs. To answer this, we first require the capacity to comprehensively detect moRNAs that is hindered by the generally low levels of their expression. Combining CAGE-seq and small RNA-seq prediction can further strengthen moRNA detection, as we detected similar number of moRNAs in zebrafish as in human (22), even though the number of annotated pre-miRNAs in human being almost 3-fold larger. Evidence for presence of moRNAs in homologous pre-miRNAs in *Ciona*, zebrafish and human indicates that the underlying moRNA biogenesis is evolutionarily conserved and may indicate a fundamental process of generation of a non-coding RNA class, which remains poorly understood. The data and annotation we have generated in this work provide a valuable resource for future investigation of these phenomena.

## AVAILABILITY

Processed data (CAGE-seq, RNA-seq and histones ChIP-seq) can be downloaded from http://promshift.genereg.net/zebrafish/. Alternatively, it can be viewed on UCSC genome browser http://www.genome.ucsc.edu/goldenPath/customTracks/custTracks.html#Zebrafish.

## Supplementary Material

SUPPLEMENTARY DATA
