# Supplementary material for: Transcriptional, post-transcriptional and chromatin-associated regulation of pri-miRNAs, pre-miRNAs and moRNAs
Source: Nucleic Acids Res. 2015 Dec 15;44(7):3070–81. doi: 10.1093/nar/gkv1354 (PMC4838339; doi:10.1093/nar/gkv1354)
Supplement: SUPPLEMENTARY DATA [file supp_44_7_3070__index.html]

Transcriptional, post-transcriptional and chromatin-associated regulation of pri-miRNAs, pre-miRNAs and moRNAs — SUPPLEMENTARY DATA 

# Transcriptional, post-transcriptional and chromatin-associated regulation of pri-miRNAs, pre-miRNAs and moRNAs

## SUPPLEMENTARY DATA

- SUPPLEMENTARY DATA
- SUPPLEMENTARY DATA
- SUPPLEMENTARY DATA
