# Supplementary material for: Transcriptional, post-transcriptional and chromatin-associated regulation of pri-miRNAs, pre-miRNAs and moRNAs
Source: Nucleic Acids Res. 2015 Dec 15;44(7):3070–81. doi: 10.1093/nar/gkv1354 (PMC4838339; doi:10.1093/nar/gkv1354)
Supplement: SUPPLEMENTARY DATA [file supp_gkv1354_nar-01433-h-2015-File008.doc]

# Supplementary Figure Legends

**Figure S1**. Schematic representation of CAGE tags and a transcript cluster (TC). **(A)** Start position of CAGE tags mapped to the genome define start of CAGE tags start sites (CTSSs) (vertical red bar). The CTSS (+1) and the immediate upstream (-1) nucleotide define the initiator sequence, which are highlighted in blue. Vertical height of the CTSS bar represents relative frequency used to quantify expression level. CTSSs overlapping within 20 bases are clustered and form a transcript cluster (TC).

**Figure S2**. Genomic architecture of miR-9 primary transcripts in zebrafish and human. **(A-F)** Pri-miRNA transcripts of *miR-9-(1/4/5)* are annotated in Ensembl (red) and pri-miRNA transcripts of *miR-9-(3/6/7)* are assembled by RNA-seq (black bars). CAGE tags from zebrafish Prim6 stage are shown in red (forward strand) and blue (reverse strand) along with H3K4me3 (Prim6 stage) and H2A.Z (30% Epiboly) tracks. **(G-H)** Annotated host transcripts of human MIR9-1/3 with overlaid histone modification (H3K4me3 and H3K27ac) tracks from Encode cell lines.

**Figure S3**. Design of *in situ* probe for each miR-9 pri-miRNA and their expression patterns. **(A)** Expression profiling of miR-9 primary transcripts determined by CAGE-seq reveals miR-9 pri-miRNAs are zygotically initiated transcripts. X-axis represents the developmental stages analyzed. Y-axis represents the expression level. Expression profiling. **(B)** Schema describing transcripts organization at miR-9 loci. Position of CAGE tags (prim6 stage) are indicated by a green peaks. Pre-miRNAs are represented by a small hairpin and exons of pri-miRNA transcripts by rectangular red boxes. Positions of the ISH probes designed are represented by rectangular blue boxes. **(C)** Expression pattern of miR-9 pri-miRNA transcripts and mature miR-9 LNA probe at 48 hpf, as determined by in situ hybridization revealed using NBT/BCIP (blue) and shown in lateral views.

**Figure S4**. Illustrative examples of sharp and broad pri-miRNA promoters. **(A)** Expression levels of pri-miRNAs and 1000 randomly selected coding genes obtained by summing the expression level of TCs in the defined promoter region. Y-axis represents expression level as measured by log2 (tpm). **(B)** Maternally inherited miR-17-92 cluster pri-miRNA use TSS from fixed nucleotide giving rise to sharp promoter. Black arrowhead points to the CAGE tag that defines dominant TSS. Pri-miRNA of miR-(363/19c/20b/18c) has multiple CAGE peaks (pointed by blue and red arrows head) spanning a larger region that gives rise to broad promoter. **(C)** Frequency of AA/AT/TA/TT (W-box) dinucleotides around 100 bases upstream and 300 bases downstream of pri-miRNA TSSs reveal an enrichment of W-box at correct position in sharp promoters.

**Figure S5**. Chromatin signatures at pre-miRNAs in human and zebrafish. **(A-D)** Alignment of average H3K4me3 **(A)**, H3K9ac **(B)**, H2A.Z **(C)** and H3K27ac **(D)** signals along the 5’-ends of pre-miRNAs on human ESC cell lines reveal an enriched peak at their 5’-ends. Y-axis indicates the average signals and X-axis indicates 2 KB region around pre-miRNAs. **(E-H)** Alignment of average H3K4me3 (**E**; 512cells stage), H3K4me3 (**F**; Prim6 stage), H2A.Z (**G**; Dome-30%Epiboly stage) and RNAPII (**H**; Dome-30%Epiboly stage) signals on pre-miRNAs based on overlapping CpG islands. Pre-miRNAs overlapping CpG islands have higher enrichment of signals. **(I)** Alignment of H3K4me3 signals on pre-miRNAs that have independent (with respect to pri-miRNA) H3K4me3 peaks or common H3K4me3 peaks (extending all the way from pri-miRNAs to pre-miRNAs) have different enrichment patterns.

**Figure S6**. Identification of CAGE tags and flanking small RNAs at Drosha cleavage sites. **(A-C)** Illustrative examples of CAGE-seq detected Drosha processing events and the resulting moRNAs. Horizontal black bars represent annotated mature miRNAs and blue bars represent pre-miRNAs. CAGE tags are represented by red vertical lines. Small RNA reads mapped to mature miRNAs are represented by red and moRNAs are represented by blue vertical blocks. **(D)** Alignment of sequences based on position of CAGE tags detected at Drosha cleavage site. Sequence logo enriched at Drosha cleavage sites is different from the logo of pri-miRNA initiators. **(E)** Overlap of CAGE-seq detected 3p-arm processing and small RNAs detected moRNAs on the set of pre-miRNA analyzed. Small RNAs detected had a minimum threshold of 1 read and 2 reads during Prim6 stage. **(F)** Detection of Drosha cleavage site (pointed by arrow) on Ensembl predicted pre-miRNA can be used as predictive tool to select true miRNA candidates. Pre-miRNA has enriched H3K4me3 signals and CAGE-seq-detected pri-miRNA in the upstream region.
