# Supplementary material for: Transcriptional, post-transcriptional and chromatin-associated regulation of pri-miRNAs, pre-miRNAs and moRNAs
Source: Nucleic Acids Res. 2015 Dec 15;44(7):3070–81. doi: 10.1093/nar/gkv1354 (PMC4838339; doi:10.1093/nar/gkv1354)
Supplement: SUPPLEMENTARY DATA [file supp_gkv1354_nar-01433-h-2015-File009.pdf]

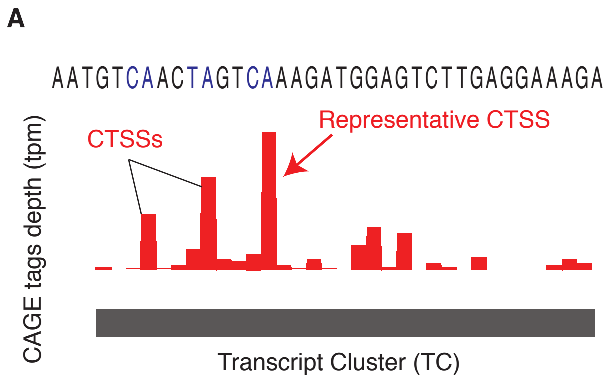

Figure S1. Schematic representation of CAGE tags and transcript cluster (TC). **(A)** Start position of CAGE tags mapped to the genome define start of CAGE tags start sites (CTSSs) (vertical red bar). CTSS and the immediate upstream (-1) nucleotide define the initiator sequence, which are highlighted by blue. Vertical height of the CTSS bar represents relative frequency that is used to quantify expression level. CTSS overlapping within 20 bases are clustered and form a transcript cluster (TC).

Figure Supplemental 1

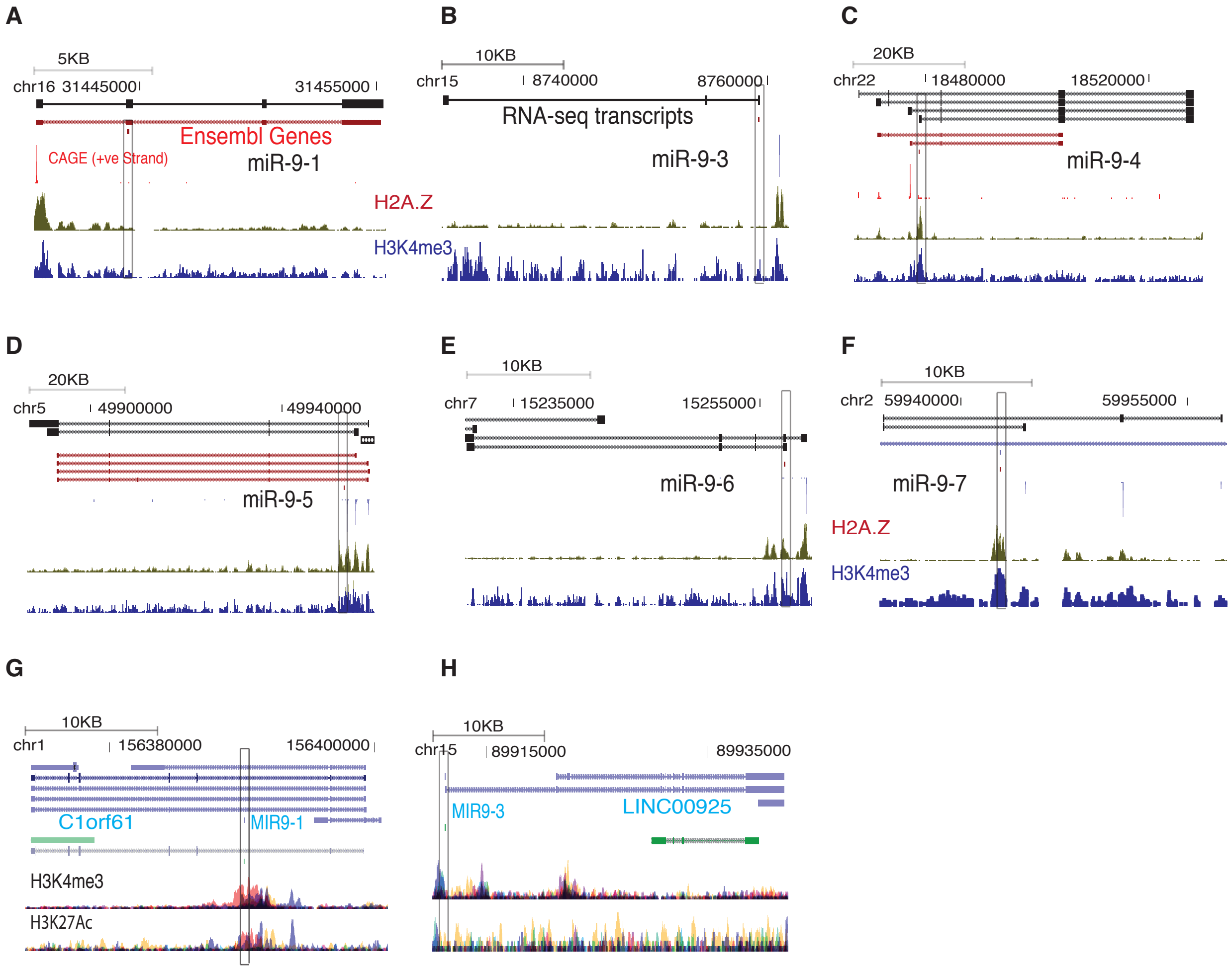

Figure S2. Genomic architecture of miR-9 primary transcripts in zebrafish and human. **(A-F)** Pri-miRNA transcripts of miR-9-(1/4/5) are annotated in Ensembl (red) and pri-miRNA transcripts of miR-9-(3/6/7) are assembled by RNA-seq (black bars). CAGE tags from prim6 stage are shown in red (forward strand) and blue (reverse strand) along with H3K4me3 (Prim6 stage) and H2A.Z (30% Epiboly) tracks. **(G-H)** Annotated host transcripts of MIR9-1/3 with overlaid histone modification (H3K4me3 and H3K27ac) tracks from Encode cell lines.

Figure Supplemental 2

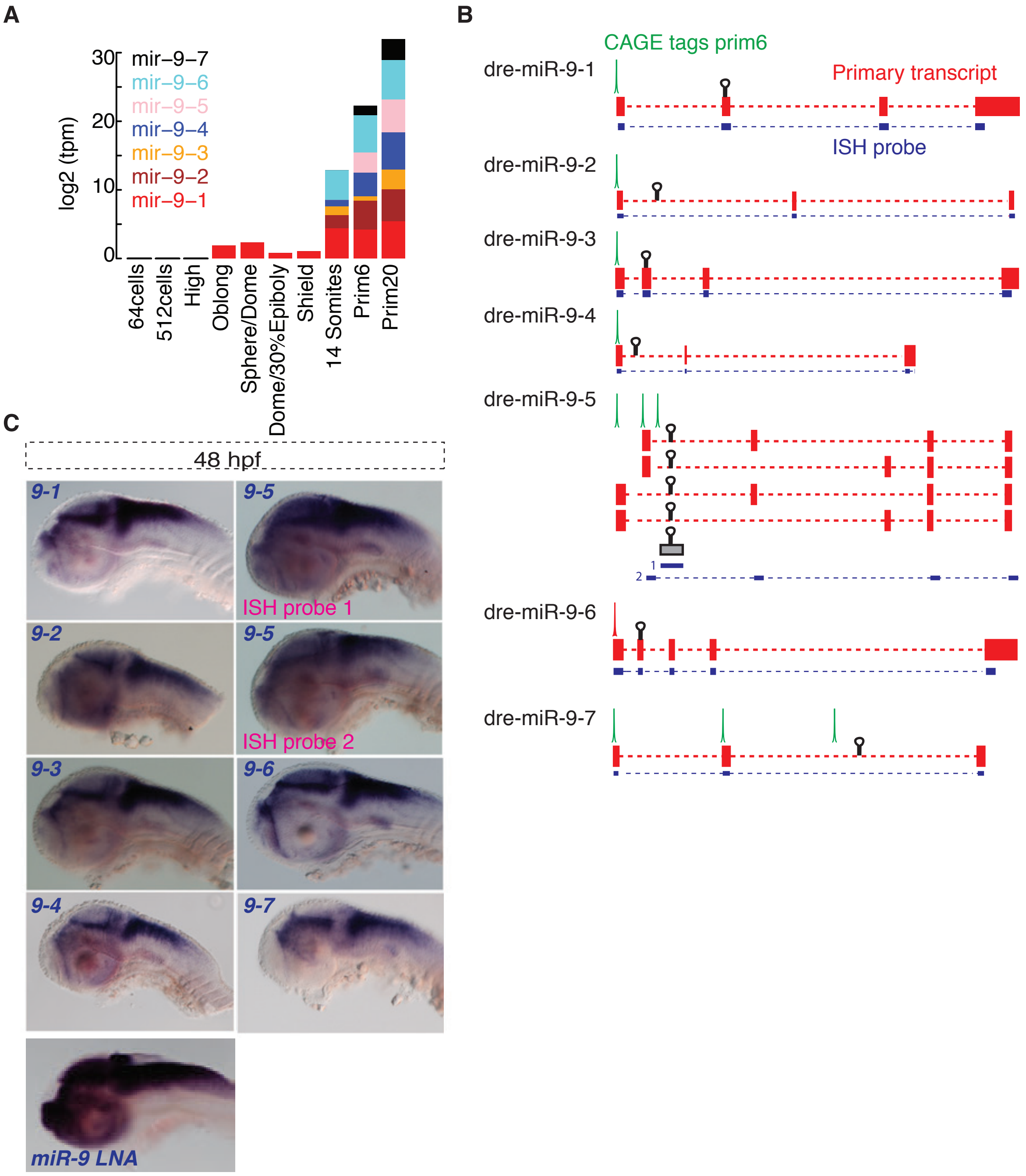

Figure S3. Design of in situ probe for each miR-9 pri-miRNA and their expression patterns. **(A)** Expression profiling of miR-9 primary transcripts determined by CAGE-seq reveals miR-9 pri-miRNAs are zygotically initiated transcripts. X-axis represents the developmental stages analyzed. Y-axis represents the expression level. Expression profiling. **(B)** Schema describing transcripts organization at miR-9 loci. Position of CAGE tags (prim6 stage) are indicated by a green peaks. Pre-miRNAs are represented by a small hairpin and exons of pri-miRNA transcripts are represented by rectangular red boxes. Positions of the designed ISH probes are represented by rectangular blue boxes. **(C)** Expression pattern of miR-9 pri-miRNA transcripts and mature miR-9 LNA probe at 48 hpf, as determined by in situ hybridization revealed using NBT/BCIP (blue) and shown in lateral views.

Figure Supplemental 3

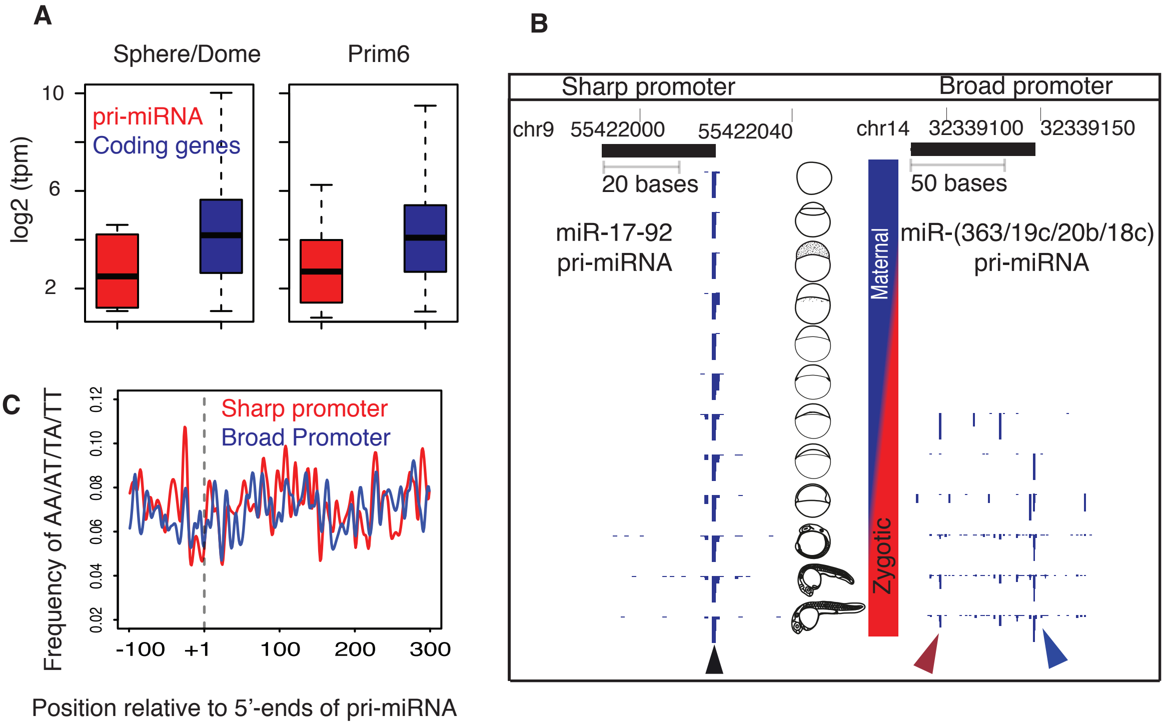

Figure S4. Expression levels of pri-miRNAs promoters and illustrative examples of sharp and broad promoters. **(A)** Expression level of pri-miRNAs and 1000 randomly selected coding genes by summing the expression level of CAGE TCs in the defined promoter region. Y-axis represents expression level in log2 (tpm). **(B)** Maternally inherited miR-17-92 cluster pri-miRNA use TSS from fixed nucleotide giving rise to sharp promoter. Black arrowhead points to the CAGE tag that defines dominant TSS. Pri-miRNA of miR-(363/19c/20b/18c) has multiple CAGE peaks (pointed by blue and red arrows head) spanning a larger region that gives rise to broad promoter. **(C)** Frequency of AA/AT/TA/TT (W-box) dinucleotides around 100 bases upstream and 300 bases downstream of pri-miRNA TSSs reveal an enrichment of W-box at correct position in sharp promoters.

Figure Supplemental 4

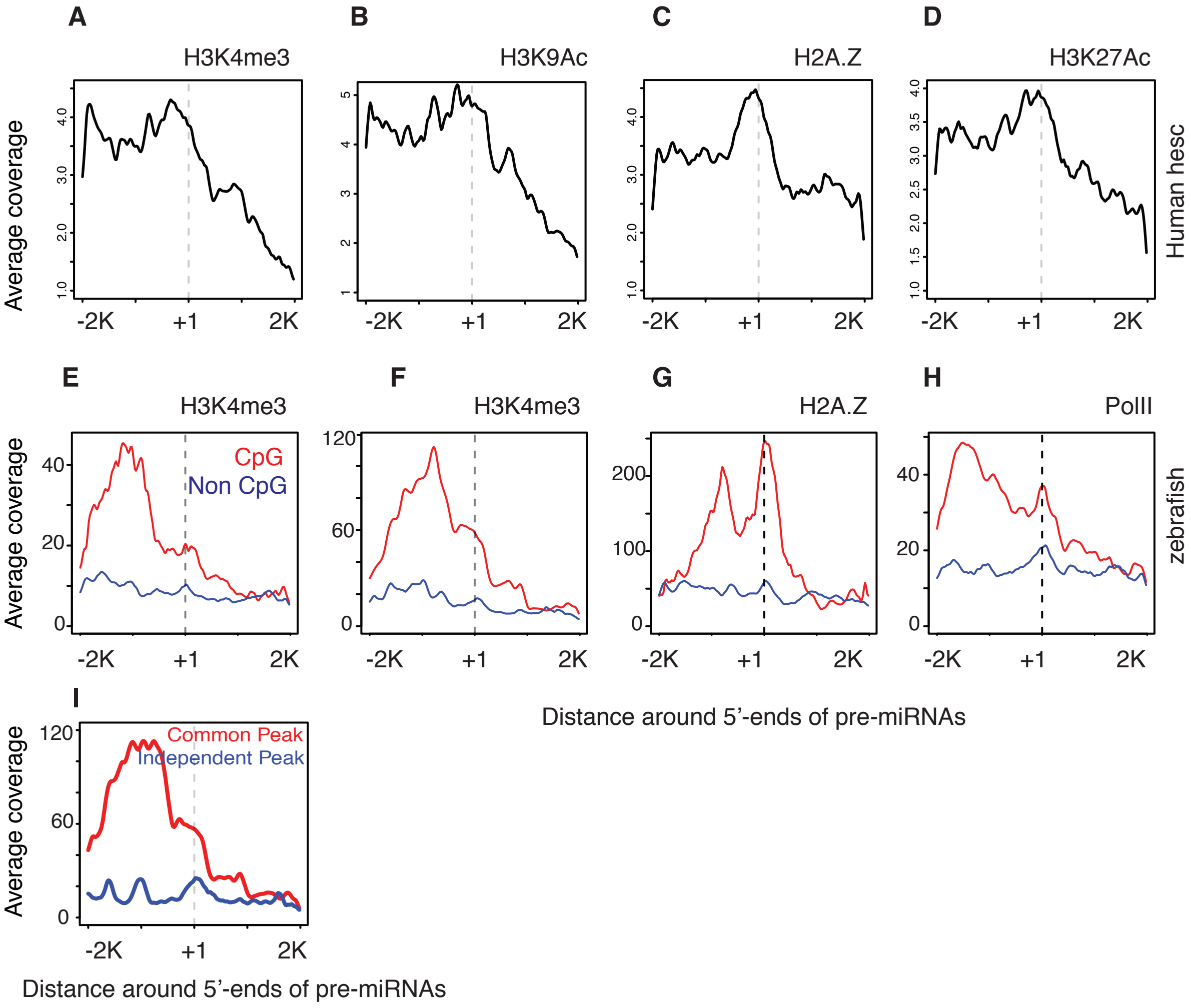

Figure S5. Chromatin signatures at pre-miRNAs in human and zebrafish. **(A-D)** Alignment of average H3K4me3 **(A)**, H3K9ac **(B)**, H2A.Z **(C)** and H3K27ac **(D)** signals along the 5'-ends of pre-miRNAs on human ESC cell lines reveal an enriched peak at their 5'-ends. Y-axis indicates the average signals and X-axis indicates 2 KB region around pre-miRNAs. **(E-H)** Alignment of average H3K4me3 (**E;** 512cells stage), H3K4me3 (**F;** Prim6 stage), H2A.Z (**G;** Dome-30%Epiboly stage) and RNAPII (**H;** Dome-30%Epiboly stage) signals on pre-miRNAs based on overlapping CpG islands show enriched signals in those overlapping with CpG islands. **(I)** Pre-miRNAs with independent (with respect to pri-miRNA) H3K4me3 peaks and common H3K4me3 peaks (extending all the way from pri-miRNAs to pre-miRNAs) have different enrichment patterns.

Figure Supplemental 5

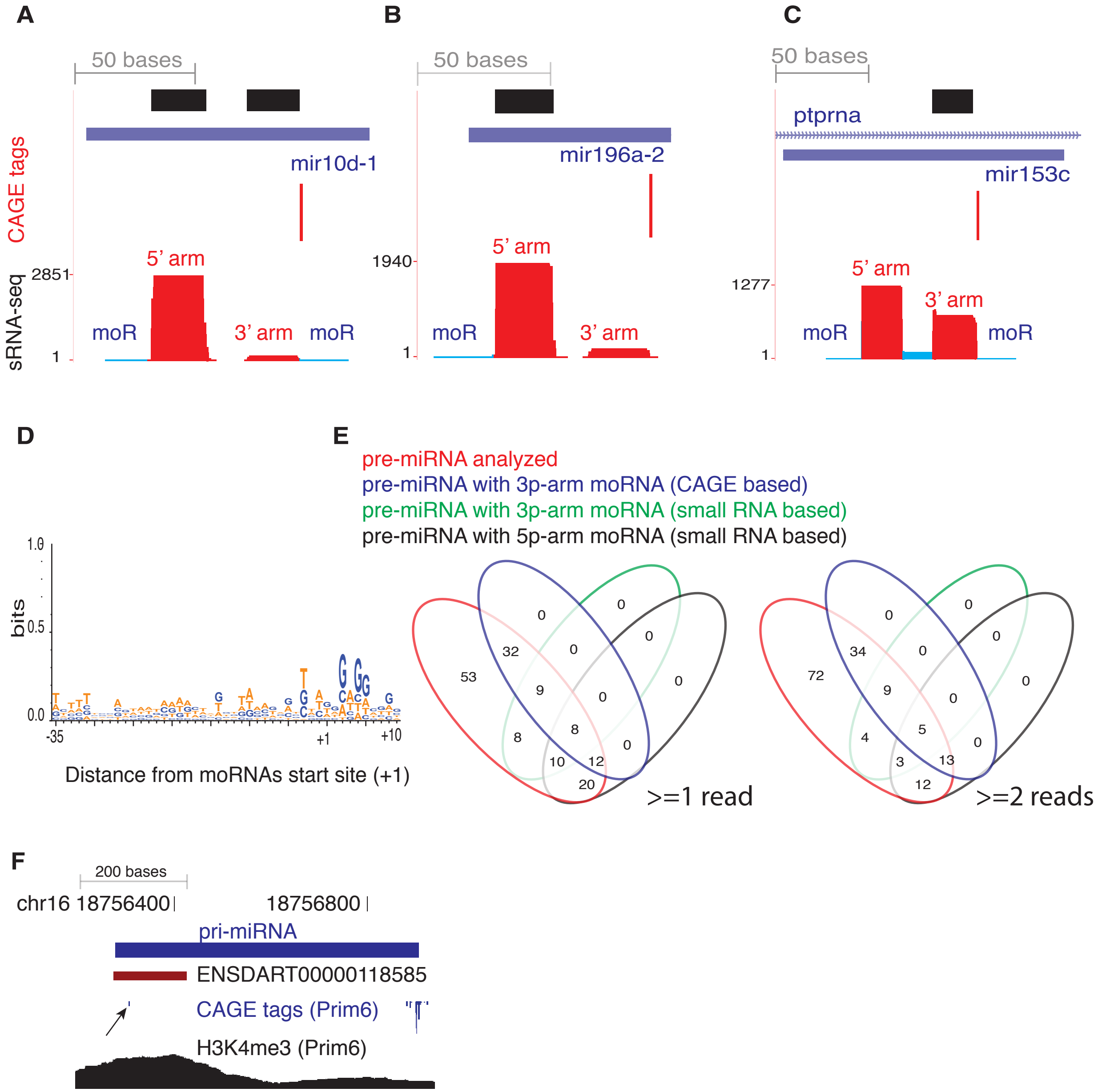

Figure S6. Identification of CAGE tags and flanking small RNAs at Drosha cleavage site. **(A-C)** Illustrative examples of CAGE-seq detected Drosha processing events and emanating moRNAs. Horizontal black bars represent annotated mature miRNAs and blue bars represent pre-miRNAs. CAGE tags are represented by red vertical lines. Small RNA reads mapped to mature miRNAs and moRNAs are represented by red and blue vertical blocks, respectively. **(D)** Alignment of sequences based on position of CAGE tags detected at Drosha cleavage site. Sequence logo enriched at Drosha cleavage sites is different from the logo of pri-miRNA initiators. **(E)** Overlap of CAGE-seq detected 3p-arm processing and small RNAs detected moRNAs on the set of pre-miRNA analyzed. Small RNAs detected had a minimum threshold of 1 read and 2 read during Prim6 stage. **(F)** Detection of Drosha cleavage site (pointed by arrow) on Ensembl predicted pre-miRNA can be used as predictive tool to select true miRNA candidates. Pre-miRNA has enriched H3K4me3 signals and CAGE-seq detected pri-miRNA in the upstream region.

Figure Supplemental 6
